# Self-Efficacy as a Central Mediator of Pain, Function, and Depression: Insights of a Cross-Sectional Analysis of Depersonalized Data from the German Pain e-Registry

**DOI:** 10.3390/jcm15083061

**Published:** 2026-04-17

**Authors:** Michael A. Überall, Philipp C. G. Müller-Schwefe, Jan-Peter Jansen, Michael A. Küster, Ingo Ostgathe, Jens Kuhn

**Affiliations:** 1 IFNAP—Private Institute of Neurological Sciences, O. Meany-MDPM GmbH, Nordostpark 51, 90411 Nürnberg, Germany; 2 Interdisciplinary Center for Pain & Palliative Care Medicine, Schillerplatz 8/1, 76033 Göppingen, Germany; philipp@mueller-schwefe.com; 3 Pain Medicine Center Berlin, Schönhauser Allee 172 a, 10435 Berlin, Germany; gf@schmerzmedizin.berlin; 4 Interdisciplinary Center for Pain & Palliative Care Medicine, 53127 Bonn, Germany; drkuester@gmx.de; 5 Pain Technologies and Clinical Monitoring GmbH, Miesbacher Str. 4, 83703 Gmund, Germany; ingo.ostgathe@dgschmerzmedizin.de; 6 Department of Psychiatry and Psychotherapy, Faculty of Medicine, University Hospital Cologne, 50937 Cologne, Germany; j.kuhn@alexianer.de; 7 Alexianer Hospital Cologne, Alexianer Köln GmbH, Kölner Strasse 64, 51149 Köln, Germany

**Keywords:** chronic pain, depression, self-efficacy, pain disability, mediation analysis, antidepressants, German pain e-Registry

## Abstract

**Background:** Depression is highly prevalent among individuals with chronic pain and strongly impacts pain intensity, psychological functioning, and health-related quality of life. Self-efficacy has emerged as a potentially modifiable resilience factor within this interplay, yet large-scale real-world evidence integrating self-efficacy into multidimensional pain–depression models remains limited. **Methods:** This cross-sectional registry-based analysis evaluated standardized patient-reported measures from chronic pain patients enrolled in the German Pain e-Registry. All variables were directionally harmonized and transformed into standardized deviation scores (hSDSs) relative to patients without depression. Group-level hSDS profiles for five DASS-21 depression severity strata (none, mild, moderate, severe, extreme) were compared across pain intensity, disability, psychological well-being, affective pain processing, quality of life, neuropathic pain features, and pain-related self-efficacy (PSEQ). Correlations and exploratory principal component analysis (PCA) were used to assess multivariate structure. PCA-informed path models were estimated to evaluate directional relationships between pain, function, depression, and self-efficacy. All directional and mediation models represent exploratory, theory-informed statistical frameworks and do not imply causal or mechanistic relationships. **Results:** Across all domains, hSDS values increased monotonically with depression severity, while self-efficacy showed the strongest inverse gradient. Exploratory PCA revealed a dominant severity component explaining most variance and a secondary affective–self-efficacy axis, supporting the conceptual separation between functional–physical and psychological–affective symptom clusters. In the bottom-up path model (pain → function → self-efficacy → depression), self-efficacy showed the largest indirect statistical contribution within the proposed path models, and the model explained 55% of depression variance (R^2^ = 0.55). In the top-down model (depression → affective pain → self-efficacy → pain), 45% of pain intensity variance was explained (R^2^ = 0.45), again with self-efficacy as a central mediating construct. Associations remained robust after adjustment for age, sex, and BMI, as well as during sensitivity analyses. **Conclusions:** This large real-world cohort demonstrates a highly coherent pattern of associations across biopsychosocial domains and highlights pain-related self-efficacy as a central statistical construct linking pain, functional impairment, and depressive symptom burden within the applied exploratory models. The findings suggest that self-efficacy occupies a key position in the interplay between pain and mood, and that pharmacological and non-pharmacological treatments traditionally used in chronic pain management may be associated with changes in this construct. Importantly, all directional and mediation analyses are exploratory and do not imply causal or mechanistic relationships. Therapeutic strategies aimed at enhancing self-efficacy may therefore represent promising targets for future research within multimodal pain management frameworks.

## 1. Introduction

Chronic pain, defined as pain persisting or recurring for more than three months, is a highly prevalent and disabling condition affecting approximately 20–30% of adults worldwide [1,2]. It imposes a substantial individual and societal burden, leading to reduced quality of life, loss of productivity, and elevated health care utilization and represents one of the leading causes of global disability and loss of productivity [3,4]. Beyond its physical dimension, chronic pain is a complex biopsychosocial phenomenon involving neurophysiological, cognitive, and emotional mechanisms that interact in dynamic and reciprocal ways [5]. Among these, depressive symptoms are particularly common, occurring in up to 40% of patients with chronic pain [6,7,8]. The co-occurrence of pain and depression amplifies disability and impairs treatment response, constituting one of the most clinically relevant yet challenging comorbidities in pain medicine [9,10].

The general association between pain and depression is bidirectional. Pain contributes to the development and persistence of depressive symptoms through physical limitations, disrupted sleep, social withdrawal, and neurochemical dysregulation [11,12,13]. Conversely, depression enhances pain perception by altering descending inhibitory control, increasing pain-related attention as well as catastrophizing, and diminishing coping capacity [14,15]. This interplay reflects a mutual maintenance model, in which emotional distress and somatic symptoms reinforce each other over time [16,17]. Understanding the precise nature and magnitude of these interactions is essential to inform targeted interventions and optimize multimodal pain management.

A growing body of evidence indicates that the link between pain and depression is mediated by functional impairment and psychological adjustment factors, including fear avoidance, catastrophizing, and particularly self-efficacy—the belief in one’s ability to exert control over one’s situation and behavior [18,19,20,21,22]. The concept of pain-related self-efficacy is central to modern cognitive-behavioral pain models, which emphasize active coping and patient empowerment [23,24]. Higher self-efficacy has been consistently associated with lower pain intensity, reduced emotional distress, and better functional outcomes, whereas low self-efficacy predicts chronicity, disability, and poor treatment adherence [25,26,27]. Accordingly, improving self-efficacy is a key target of contemporary psychological and interdisciplinary pain interventions.

Despite its theoretical and clinical relevance, few large-scale studies have systematically analyzed self-efficacy as part of a multidimensional model linking pain, function, and depression in real-world settings. Experimental and smaller cohort studies suggest that pain-related self-efficacy mediates the relationship between depressive symptoms and functional outcomes [28,29,30], and that improvements in self-efficacy predict better long-term adjustment and treatment response [31,32]. However, these studies have generally been limited by small sample sizes, single-instrument assessments, or cross-sectional designs, which preclude a comprehensive understanding of bidirectional interactions among these domains. Moreover, little is known about how the magnitude of these interrelationships evolves with increasing depression severity or how they translate into integrated patterns at the population level.

To address these gaps, large-scale registry data offer unique opportunities as they allow, among others, the evaluation of multifactorial interrelationships (e.g., pain–mood) under real-world conditions [33]. The German Pain e-Registry (GPeR) represents one of the most extensive databases of patient-reported outcomes (PROs) in chronic pain, capturing multidimensional information on pain, function, mood, and treatment characteristics from hundreds of thousands of patients across specialized and primary care settings in Germany. Using this infrastructure, it becomes possible to harmonize data across diverse validated instruments, model cross-domain dependencies, and test competing causal pathways at scale.

### Study Rationale

The present study aimed to systematically examine the interrelationships between pain intensity, functional impairment, depressive symptomatology, and pain-related self-efficacy in a large real-world cohort of patients with chronic pain documented in the GPeR.

Recognizing that pain and depression often interact bidirectionally, we sought to clarify the relative strength and direction of these associations within a biopsychosocial framework. Specifically, this analysis pursued four complementary objectives:

1. Quantification of domain-specific effects—to determine how standardized deviations (SD) in pain, function, quality of life, and self-efficacy vary across clinically defined levels of depressive symptom severity.
2. Normalization across heterogeneous instruments—to harmonize and directly compare outcomes from multiple validated self-report measures, ensuring that higher SD scores (SDS) uniformly represent greater impairment across physical, psychological, and social domains.
3. Identification of structural relationships—to explore domain clustering through hierarchical correlation and principal component analyses, thereby revealing latent patterns that characterize the biopsychosocial organization of chronic pain.
4. Modeling of causal directionality—to contrast bottom-up (pain → function → depression) and top-down (depression → affective pain → pain intensity) pathways using mediation and regression modeling, with particular attention to the mediating role of self-efficacy as a central adaptive construct.

Based on existing evidence linking self-efficacy to coping, resilience, and treatment response, we hypothesized that:

1. Most pain- and function-related SDS would increase systematically with depression severity, whereas self-efficacy would inversely decrease.
2. The relationship between pain and depression would be primarily mediated by functional impairment and psychological factors (bottom-up model).
3. Depression would exert a secondary, yet significant, influence on pain via affective pain processing (top-down model).

## 2. Methods

### 2.1. Study Design and Data Source

To empirically test these hypotheses, we performed a cross-sectional, registry-based exploratory analysis of routinely collected patient-reported data from the German Pain e-Registry (GPeR). The GPeR represents one of the largest real-world data infrastructures for chronic pain care in Europe, capturing cross-sectional as well as longitudinal information from specialized pain centers, general practitioners, and interdisciplinary outpatient programs across Germany. The registry operates in compliance with the EU General Data Protection Regulation (GDPR), Article 9(2)(j), for scientific use of pseudonymized health data and is operated under the joint governance of the Center of Excellence for Health Care Research at the Institute of Neurological Sciences and the German Pain League (Deutsche Schmerzliga, DSL), both of which are independent, non-commercial and non-governmental organizations which represent the interests of pain specialists and pain patients. The GPeR functions as a digital health infrastructure that links patients, outpatient pain clinics, and multidisciplinary care networks through standardized documentation processes.

The GPeR collects data from patients receiving specialized pain therapy, integrating both clinical metadata and patient-reported outcome measures (PROMs). However, in the present analysis, only patient-reported measures (PRMs) were used. This focus ensures that the results reflect the subjective experience and perceived functional impact of chronic pain without the potential bias introduced by physician-rated metrics. PRMs are captured directly through a secure digital interface prior to routine consultations. Patients complete individually selected and scientifically validated questionnaire autonomously at home or in pain centers using tablet devices, guided by adaptive logic that minimizes missing entries.

Once entered, data undergo real-time plausibility checks to identify incomplete or inconsistent responses. Healthcare professionals (HCPs) review and verify demographic or diagnostic information. Verified data are then locked and released to the central registry database, where further automated quality control is performed before the data become available for aggregated analyses.

At the registry level, continuous quality assurance procedures are implemented, including validation of internal consistency, range checks, and periodic audits of data completeness. The overall completeness rate for PRM fields exceeds 95%, reflecting the system’s design to capture standardized, high-quality data at scale. Prior to any research use, mirrored datasets undergo an additional round of plausibility testing and anonymization, ensuring compliance with both the European Union’s General Data Protection Regulation (GDPR) as well as German data privacy standards.

### 2.2. Patient Population and Inclusion Criteria

The present analysis included depersonalized raw data from adult patients (≥18 years) with a diagnosis of chronic pain persisting for at least three months and documented participation in the GPeR between January 2015 and December 2024. Chronic pain was defined in accordance with the International Association for the Study of Pain (IASP) criteria [1]. Exclusion criteria comprised incomplete baseline PRM data, implausible demographic information, or missing DASS-21 scores necessary to categorize depression severity. Data from patients with acute pain episodes only and/or malignancy-related pain were excluded to enable the aggregation of a harmonized cohort of patients with chronic non-cancer pain.

### 2.3. Instruments and Variables

All instruments used in this study are components of the German Pain Questionnaire (Deutscher Schmerzfragebogen), which is the standardized assessment framework jointly endorsed by the German Pain Association and the German Pain League and recommended under the German National Quality Assurance Agreement for Specialized Pain Therapy (§135 SGB V) [33]. The questionnaire integrates psychometrically validated self-report instruments covering essential biopsychosocial dimensions of chronic pain.

#### 2.3.1. Pain Intensity, Disability/Interference with Daily Life and Functional Capacity

Pain intensity was measured using 100 mm Visual Analogue Scales (VASs)—ranging from “no pain” (0) to “worst pain imaginable” (100)—for the lowest (LPI), average (API), and highest (HPI) pain experienced over the preceding 24 h [33]. These values were used to compute a 24-h Pain Intensity Index (PIX), defined as the arithmetic mean of LPI, API, and HPI.

Pain-related disability was assessed with the modified Pain Disability Index (mPDI) [34], which evaluates interference with seven domains of daily life: (1) household and family activities, (2) leisure and relaxation, (3) social activities, (4) occupational functioning, (5) essential daily activities, (6) sleep, and (7) enjoyment of life. Each domain is rated on a 100 mm VAS ranging from “no disability” (0) to “worst disability imaginable” (100). In addition to the individual scores for the seven domains (mPDI1-7), the overall mPDI score is calculated as the arithmetic mean of the individual subdomain values and is accepted as a composite measure for the overall pain-related disability in daily life activities.

Pain-related interference with daily life was assessed using core components of the Chronic Pain Grade Scale (CPGS), a multidimensional seven-item instrument that grades chronic pain [35]. For the present analysis, emphasis was placed on items 4–7, which assess the degree to which pain limits daily life activities. Item 4 measures the number of days within the past three months during which usual activities were prevented because of pain, capturing the temporal extent of disability. Items 5–7 assess interference in the domains of general daily activities, social and family/leisure activities, and work or household activities, respectively, using 100 mm VASs (0 = “no interference”, 10 = “extreme interference”).

Functional capacity was assessed using the Functional Questionnaire Hannover Back Pain (FFbH-R), a validated 12-item self-report instrument measuring limitations in activities of daily living due to back pain [36]. Each item is rated on a 3-point scale (0 = “without difficulty,” 1 = “with some difficulty,” 2 = “not possible/only with help”), and responses are transformed into a percentage functional capacity score ranging from 0% (maximum limitation) to 100% (full functional ability). The FFbH-R is routinely implemented in the German Pain e-Registry to evaluate functional impairment in patients with chronic pain, predominantly but not exclusively back pain. Established interpretive thresholds suggest a score ≥ 80% indicates good functional capacity while scores < 60% indicate substantial functional limitation, and values in between reflect moderate impairment.

#### 2.3.2. Pain Phenotype

Pain phenotype was characterized using the short form of the PainDETECT Questionnaire (PDQ7) [37]. It consists of seven sensory-descriptor items (e.g., burning, tingling, allodynia, numbness, and hyperalgesia, etc.), which are individually scored on an adjectival scale from 0 = “never” to 5 = “very strongly”. The total score (0–35) classifies pain as “likely nociceptive” (<10), “mixed” (10–17), or “probably neuropathic” (≥18).

#### 2.3.3. Quality of Life

Health-related quality of life was assessed using the VR-12, a 12-item self-report instrument derived from the longer VR-36 and designed to provide a concise measure of overall physical and mental health status, [38] producing physical (PCS) and mental (MCS) component scores standardized to a mean of 50, with higher scores indicating better health status.

Pain-related quality of life restrictions were evaluated with the Quality-of-Life Impairment by Pain Inventory (QLIP) [33], which quantifies how pain affects physical, emotional, and social well-being. QLIP sum scores range between 0 (“worst impairment possible”) to 40 (“no impairment”), and QLIP scores ≤ 20 indicate substantial impairment.

#### 2.3.4. Psychological and Affective Factors

The Depression Anxiety Stress Scales—21-item version (DASS-21) was employed to determine the severity of depressive symptoms, which served as the primary stratification variable for all analyses [39]. The DASS-21 is a self-report instrument measuring the core symptoms of depression, anxiety, and stress on three 7-item subscales [39]. Each item is rated on a 4-point Likert severity/frequency scale ranging from 0 = “did not apply to me at all” to 3 = “applied to me very much or most of the time”, yielding subscale scores from 0 to 21, with higher scores indicating stronger severity of symptoms. In accordance with official German-language scoring guidelines and recommendations of the German Pain Association, the severity of the depressive burden was categorized as none (0–4), mild (5–6), moderate (7–10), severe (11–13), or extreme (≥14). The DASS-21 is the consensus standard recommended by the German pain expert and patient associations for psychological screening in chronic pain documentation and routinely integrated into the GPeR as the standardized tool for emotional assessment in specialized pain therapy and quality assurance (§135 SGB V) and was therefore chosen as the core instrument for this registry analysis.

Pain perception was measured using the Pain Perception Scale (PPS) developed by Geissner, which assesses the sensory-discriminative and affective-motivational dimensions of pain [40,41]. The SES consists of pain-descriptive adjectives that patients rate on a 4-point ordinal scale (1 = “not applicable”, 2 = “somewhat applicable”, 3 = “fairly applicable,” 4 = “completely applicable”) with regard to how closely these descriptors characterized their pain experience during the reference period. Separate scores are calculated for the sensory and affective subscales, with higher scores indicating stronger sensory pain sensations or more pronounced affective pain responses.

Emotional well-being was measured using the Marburg Questionnaire on Habitual Health Findings (MQHHF) [42], assessing perceived general well-being across seven items on a 5-point Likert scale (0 = “severe restriction” to 5 = “none”), which are used to calculate the actual meaningful sum value, ranging from 0 (“maximum possible impairment”) to 35 (“no impairment”). Scores ≤ 10 are considered an indication of severe impairments.

Pain-related fear/anxiety was assessed using the Fear–Avoidance Beliefs Questionnaire (FABQ), a 16-item self-report instrument measuring fear–avoidance beliefs related to physical activity and work [43]. Items are scored on a 7-point Likert scale (0 = “strongly disagree” to 6 = “strongly agree”), yielding two subscales: FABQ-Physical Activity (0–24) and FABQ-Work (0–42), with higher scores indicating stronger fear–avoidance beliefs.

#### 2.3.5. Self-Efficacy

Pain-related self-efficacy was measured with the Pain Self-Efficacy Questionnaire (PSEQ) [44], a 10-item scale assessing confidence in performing daily activities despite pain. Each item is rated from 1 (“not at all confident”) to 6 (“completely confident”), yielding a total score between 10 and 60. Higher scores indicate stronger self-efficacy. A threshold of ≤25.6 was used to define clinically significant impairment, consistent with previous research validating its predictive value for treatment outcomes.

#### 2.3.6. Demographic Variables

Age, sex, body height and weight (included to calculate body mass index, BMI) were included and served as covariates in multivariate analyses. These variables provide demographic normalization and allow evaluation of potential confounding by physiological factors.

### 2.4. Data Harmonization and Standardized Deviation Scores

Because the instruments used in this study measure diverse aspects of the pain experience—including intensity, function, affective distress, self-efficacy, and quality of life—they differ not only in scaling but also in score directionality. Some scales (e.g., PSEQ, QLIP, and the VR-12 component scores) assign higher values to better functioning or well-being, whereas others (e.g., pain intensity, mPDI, DASS-21) associate higher scores with worse symptom burden.

To enable coherent interpretation and comparability across domains, all scales were directionally aligned prior to analysis, such that higher scores consistently represented greater symptom severity, functional limitation, or psychological distress. For scales in which higher values originally indicated better outcomes, scores were algebraically inverted prior to subsequent standardization. This transformation preserved each variable’s original variance and relative ranking while ensuring that all indicators could be interpreted along a common axis (“higher = worse”).

Following directional harmonization, all continuous variables were transformed into standard deviation scores (hSDS) relative to the mean (μ) and standard deviation (σ) of the non-depressed reference group, according to the formula:

$$hSDS=\frac{X-\mu_{none}}{\sigma_{none}}$$

This normalization procedure placed all variables on a shared standardized metric, quantifying deviation from normative functioning (i.e., the average of non-depressed patients) in units of standard deviation.

Positive hSDS values therefore indicate worse-than-reference outcomes, while negative values denote better-than-reference functioning or well-being.

By anchoring the standardization to the non-depressed subgroup rather than to the total cohort, the resulting hSDS values provide a clinically meaningful reference frame that directly reflects the degree of psychological and functional deviation associated with increasing depression severity. This harmonization and normalization strategy ensured both metric comparability across heterogeneous constructs and clinical interpretability of changes across the depression continuum. Importantly, hSDS values quantify relative deviations from the non-depressed reference group and do not represent diagnostic thresholds or absolute clinical cut-off values. While this harmonization facilitates cross-domain comparability, it may attenuate scale-specific nuances and should be interpreted as a relative deviation metric rather than an absolute clinical effect size.

After hSDS computation, descriptive statistics (mean, standard deviation, 95% confidence interval) were calculated for each depression subgroup, and every single item was evaluated as well as for the whole range of parameters. To visualize deviations, hSDS means were plotted as bar and box plots. Patients exceeding clinically meaningful deterioration thresholds (SDS ≥ 1 and ≥2) were quantified to estimate population-level burden.

### 2.5. Statistical and Analytical Strategy

The analytical workflow followed an exploratory multivariate/multistage approach designed to evaluate both structure and directionality of the interrelations between pain, depression, and self-efficacy.

#### 2.5.1. Correlation and Multivariate Structure Analysis

Pairwise Pearson correlations were computed for all hSDS-transformed variables to identify patterns of shared variance across domains. Correlations of r ≥ 0.40 were considered substantial. Hierarchical cluster and principal component analysis (PCA) based on aggregated cohort means across the five levels of depression severity were conducted as exploratory methods to examine whether the empirical multivariate structure aligned with the theoretical organization of pain, function, affective disturbance and self-efficacy.

For exploratory clustering, the hSDS correlation matrix was evaluated using Ward’s minimum variance method with Euclidean distance to assess whether variables tended to group along conceptually coherent dimensions. To further characterize latent structure, a PCA was performed on standardized hSDS profiles to determine (a) whether a dominant severity component explained shared variance across domains, and (b) whether secondary components separated functional–physical, psychological–affective, and self-efficacy constructs. The resulting component structures were used to specify the directional path models (see below), ensuring that mediation analyses were grounded in observed multivariate relationships rather than imposed solely on theoretical grounds.

#### 2.5.2. PCA-Informed Path Analysis: Bottom-Up and Top-Down Frameworks

To examine potential causal directionality, two complementary path models were estimated:

(a) Bottom-up model: pain intensity → functional limitation → self-efficacy → depression;
(b) Top-down model: depression → affective pain processing → self-efficacy → pain intensity.

Model fit was assessed by explained variance (R^2^), standardized path coefficients (β), and overall significance (*p* < 0.001). These models were designed to reflect theoretical frameworks of pain–mood interaction: the bottom-up approach corresponding to functional and behavioral consequences of pain, and the top-down approach representing affective modulation of pain perception. These models were not intended to establish causality but to explore theoretically grounded directional dependencies compatible with the cross-sectional data structure.

#### 2.5.3. Control Variables and Confounding

All analyses were controlled for age, sex, and body mass index (BMI), as these demographic variables are known to influence both pain and psychological outcomes. Including them as covariates ensured that the observed associations were not artifacts of demographic skew.

#### 2.5.4. Model Performance and Robustness

Model fit and explanatory power were assessed via total R^2^, significance of indirect paths, and consistency of path coefficients across bootstrapped samples. Sensitivity analyses excluding extreme depression cases were performed to ensure the robustness of mediation results.

### 2.6. Statistical Procedures Performed

Descriptive and inferential statistical methods describe continuous variables using mean, standard deviation (SD), median, and range, as well as 95% confidence intervals (CI), and categorical variables using frequency and (adjusted) percentages. For comparative analyses, continuous variables were compared across depression severity strata using appropriate parametric methods when distributional assumptions were met. Given the large sample size and approximate normal distribution of most standardized variables, parametric comparisons were considered robust. Where appropriate, non-parametric procedures were applied. Chi-square tests were used for categorical variables. Odds ratios (OR) and relative risks (RR; each including 95% confidence intervals), Cohen’s d and the correlation coefficient Phi were calculated as effect size (ES) estimates where appropriate. Correlation analyses were performed using Pearson correlation coefficients for standardized continuous variables. Clustering procedures and principal component analysis (PCA) were conducted to explore the multivariate structure. Mediation and path analyses were estimated separately as exploratory cross-sectional models, as described above, and do not imply temporal or causal relationships. Statistical tests were performed with a two-sided significance level of 0.05, and a Bonferroni correction was used to adjust for multiplicity. Descriptive statistics, clustering procedures, and path models were reported with appropriate metrics (R^2^, β, and 95% CI). Tables and figures were created in accordance with the format of the underlying data to ensure reproducibility and interpretability across analytic stages.

### 2.7. Software and Reproducibility

Data management, descriptive and inferential analyses were performed in SPSS Statistics v29 (IBM Corp., Armonk, NY, USA). Correlational and cluster analyses were computed within SPSS using the PROCESS macro (Model 6). Mediation models (path analyses) with bootstrapped indirect effects (5000 resamples; bias-corrected 95% confidence intervals) were conducted with the lavaan package. Tables and graphs were rendered using Microsoft Excel^®^ for Microsoft 365 MSO (Version 2504 Build 16.0.18730.20122).

### 2.8. Ethics and Data Protection

This non-interventional, retrospective analysis was conducted in accordance with the ethical standards of the Declaration of Helsinki (latest revision, 2013) [45] and the applicable German and European regulations governing health-care research and data protection. Because the study was based entirely on anonymized, routinely collected data from the German Pain e-Registry (GPeR), no additional ethical approval or patient contact was required.

The GPeR operates under the joint governance of the Center of Excellence for Health Care Research at the Institute of Neurological Sciences and the German Pain League. Both organizations maintain independent steering committees responsible for scientific oversight, data governance, and ethical compliance. The study concept and the planned use of anonymized registry data were reviewed and formally approved by these committees prior to analysis.

Both patients and pain centers provided written informed consent prior to enrollment in/use of the GPeR, explicitly authorizing the anonymized use of their data for research and quality assurance purposes. No personally identifiable information was available to investigators at any stage of this analysis.

All data handling and statistical processing adhered strictly to the principles of the European Union General Data Protection Regulation (EU-GDPR, Regulation EU 2016/679) and corresponding German data protection legislation. Data were stored and processed on secure, encrypted servers located within Germany. Each data record was verified for completeness and consistency prior to inclusion, and—in compliance with the EU-GDPR and the German Federal Data Protection Act—only depersonalized, non-traceable data were mirrored, and only these anonymized data sets were used for the biometrical procedures described in this paper.

The study was registered prior to being conducted with the European Network of Centers for Pharmacoepidemiology and Pharmacovigilance (ENCePP) in the EU electronic registry of post-authorization, epidemiological and observational studies (EUPAS identifier: 1000000491).

### 2.9. Reporting and Transparency

The conduct and reporting of this study followed the STROBE (Strengthening the Reporting of Observational Studies in Epidemiology) recommendations for observational research [46]. All analyses were performed according to a pre-specified statistical analysis plan. In line with good research practice, the analytic workflow was documented in detail, including data cleaning, harmonization steps, and quality control procedures. No external funding influenced data selection, analysis, or interpretation. The corresponding author had full access to all data and final responsibility for submission.

### 2.10. Availability of Data and Materials

Data access is restricted and subject to registry governance approval. Aggregated or summary data may be made available upon reasonable request to the corresponding author and following authorization by the GPeR steering committee of the Center of Excellence for Health Care Research at the Institute of Neurological Sciences.

## 3. Results

### 3.1. Study Population

**Figure 1:**
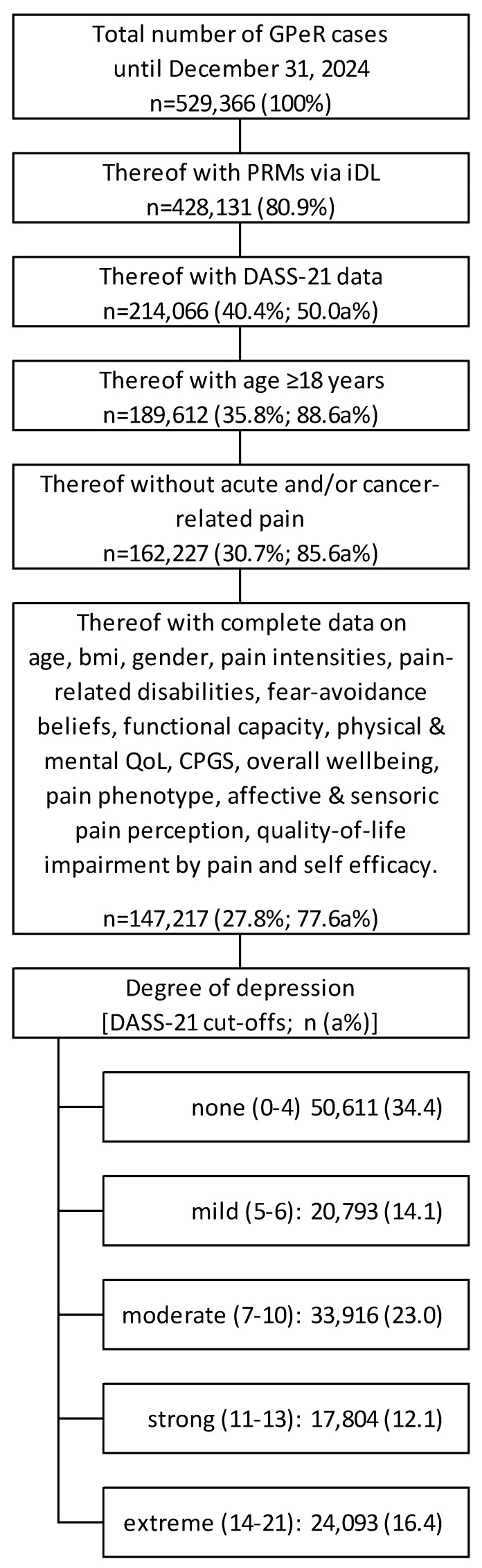
STROBE-style participant flow diagram of cohort selection and data inclusion. Depicts patient flow through the registry screening and inclusion process, from initial dataset (n = 529,366) to final analytic sample (n = 147,217).

Figure 1 presents the flow of patient data inclusion, exclusion, and stratification according to STROBE-style participant flow documentation, documenting each step from initial registry enrollment to completion of the analysis.

As of 31 December 2024, the registry comprised 529,366 treatment cases, of which 428,131 (80.9%) reported patient-reported measures (PRMs) through the electronic platform iDocLive. Among these, 214,066 (50.0% of those with PRMs) provided complete information on depressive symptom severity using the DASS-21, and 189,612 (88.6% of those with DASS-21 data) of them were adults. After excluding cases with acute and/or cancer-related pain as well as those with incomplete or missing information on essential parameters (e.g., age, gender, pain intensity, functional status, quality of life), 147,217 datasets (77.6% of eligible adults) remained and served as the analytical sample for this study.

Within this sample, the distribution across the five DASS-21 depression severity categories was as follows: none (n = 50,611; 34.4%), mild (n = 20,793; 14.1%), moderate (n = 33,916; 23.0%), strong (n = 17,804; 12.1%), and extreme (n = 24,093; 16.4%).

### 3.2. Raw Data and Harmonized Absolute/Relative Variations Across Depression Categories

Baseline demographic characteristics were broadly comparable across groups, with a mean age of 55.9 ± 15.5 years, a female proportion of 64.5% (n = 94,945), and an average BMI of 27.7 ± 6.4 kg/m^2^ (see Table 1). Variation across depression categories was modest (age: 2.7%; BMI: 5.2%; female gender: 2.4%), indicating only minimal comorbidity accumulation associated with increasing depression severity for these parameters.

**Figure 2:**
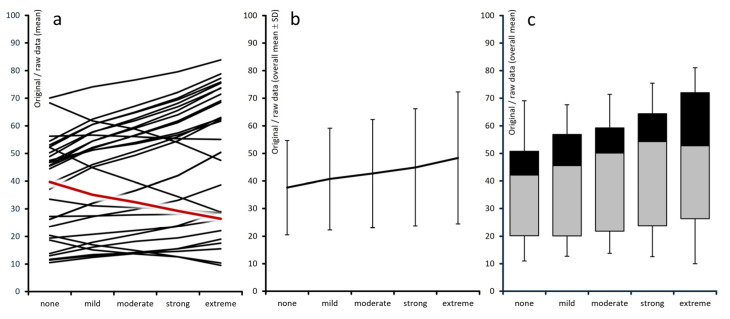
Graphical aggregation of raw values. Left panel (**a**): individual instrument values; center (**b**): aggregated domain means (standard deviation scores); right (**c**): box-and-whisker plot (boxes 25th–median–75th percentiles; whiskers: 5th and 95th percentiles).

Average raw scores for the remaining 28 pain-related parameters assessed in this study were generally representative of the patient cohort (see left section of Table 1 and Figure 2a–c); however, they exhibited substantial variability across depression categories, with a consistent pattern of deterioration corresponding to higher levels of depression. Variability ranged from 17.5% for the VR-12 Physical Component Score to 114.7% for the VR-12 Mental Component Score. Overall, 25 parameters (89.3%) displayed a variability of ≥30% across depression groups, 17 (60.7%) ≥ 50%, and five (17.9%) ≥ 80%.

**Figure 3:**
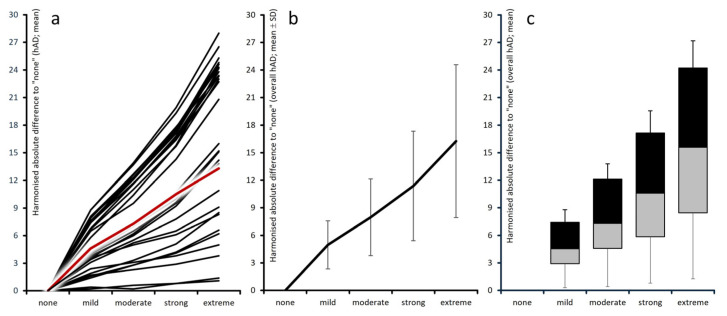
Graphical aggregation of harmonized absolute deteriorations vs. “none”. Left panel (**a**): individual instrument values; center (**b**): aggregated domain means (standard deviation scores); right (**c**): box-and-whisker plot (boxes 25th–median–75th percentiles; whiskers: 5th and 95th percentiles).

**Figure 4:**
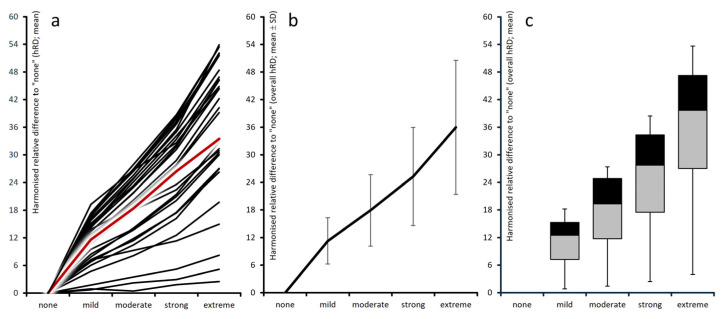
Graphical aggregation of harmonized relative deteriorations vs. “none”. Left panel (**a**): individual instrument values; center (**b**): aggregated domain means (standard deviation scores); right (**c**): box-and-whisker plot (boxes 25th–median–75th percentiles; whiskers: 5th and 95th percentiles).

Harmonized absolute differences compared with patients without depression (middle section of Table 1 and Figure 3a–c) showed a progressive deterioration: 5.0 ± 2.6 points in patients with mild depression, 8.0 ± 4.2 in those with moderate depression, 11.4 ± 6.0 in those with severe depression, and 16.3 ± 8.3 points in those with extreme depression. Harmonized relative deviations from patients without depressive DASS-21 scores (right section of Table 1 and Figure 4a–c) were on average ± standard deviation (median) 11.3 ± 5.0% (13.0) for mild, 17.9 ± 7.8% (19.9) for moderate, 25.3 ± 10.7% (28.3) for severe, and 26.0 ± 14.6% (41.2) for extreme depression. Among the 28 PRO instruments, harmonized relative deterioration for patients with extreme versus those without depressive symptomatology reached ≥30% in 22 instruments (78.5%), ≥40% in 15 (53.6%), and ≥50% in six (21.4%).

### 3.3. Harmonized Standard Deviation Scores

**Figure 5:**
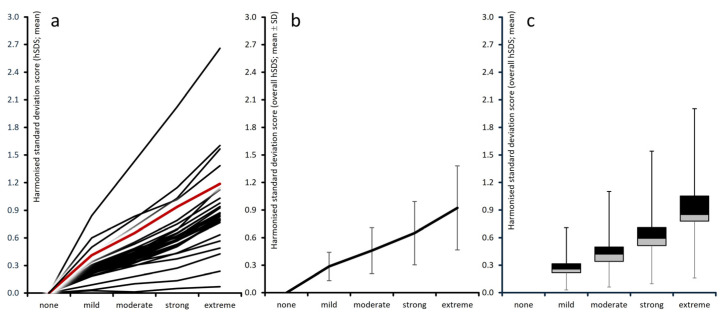
Graphical aggregation of harmonized standard deviation scores. Left panel (**a**): individual instrument values; center (**b**): aggregated domain means (standard deviation scores); right (**c**): box-and-whisker plot (boxes 25th–median–75th percentiles; whiskers: 5th and 95th percentiles).

**Figure 6:**
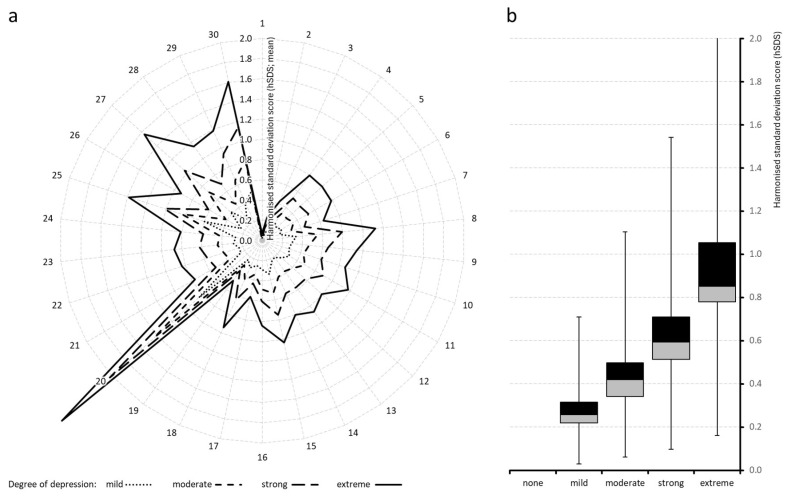
Average harmonized standardized deviation scores (hSDSs) across depression severity categories. Radar (spider) plot (**a**, **left**) showing harmonised standard deviation scores (hSDS) across 30 items, stratified by degree of depression (mild/moderate/strong/extreme) and referenced vs. none. Each axis represents one item (1–30), and the distance from the center indicates the magnitude of the hSDS (mean values). Different line styles correspond to depression severity levels. Boxplots (**b**, **right**) illustrating hSDS across four depression severity groups (mild, moderate, strong, extreme) referenced vs. none. The boxes represent the interquartile range (IQR), with the median indicated by the horizontal line, and whiskers for 5/95% percentiles showing variability.

Harmonized standardized deviation scores (hSDSs) vs. those of patients aggregated in the cohort with “no depressive symptomatology” were computed for all 28 PRMs. Table 2 summarizes the means (left), standard deviations (middle) and corresponding 95% confidence intervals (right). Figure 5a–c and Figure 6a,b show the corresponding graphs.

Across all domains, hSDS values increased monotonically with depression severity, confirming a graded, linear association between depressive symptom load and worsening of biopsychosocial function. Average ± standard deviation (median) hSDSs across all variables rose from 0.00 ± 1.00 (0.00) in the non-depressed cohort to 0.28 ± 0.15 (0.26) for patients with mild depression, 0.45 ± 0.25 (0.42) with moderate depression, 0.64 ± 0.34 (0.59) with strong depression, and 0.92 ± 0.45 (0.85) for patients in the extreme depression group. Significance vs. “no depression” was <0.001 for all other cohorts, and corresponding effect sizes increased from 0.288 for “mild”, to 0.465 for “moderate”, 0.662 for “strong”, and up to 0.944 for “extreme depression” (each vs. “none”).

Highest hSDS vs. none were found for all depression cohorts for the mental component score of the VR-12 (which increased from 0.84 for “mild depression” to 1.43 and 2.02 for “moderate and strong depression” and to 2.66 for “extreme depression”), followed by the quality-of-life impairment by pain QLIP scores (with hSDS increasing from 0.50 to 1.60), the affective pain perception SES-A (0.42 to 1.57), and overall well-being, assessed with the MQHHF (0.60 to 1.38).

All pain-related indices showed progressive deterioration with increasing depression. The 24-h Pain Intensity Index (PIX) increased from 0.00 to 0.80 hSDS, while the subcomponents for the lowest, average, and highest pain intensities ranged between 0.63 and 0.79 hSDS. Even at mild depression levels, pain intensities were already elevated (0.18 to 0.22 hSDS), underscoring an early coupling of mood and nociceptive experience.

The Modified Pain Disability Index (mPDI) and its subscales demonstrated pronounced increases, reflecting greater functional impairment. The average mPDI score rose by 1.12 hSDS in the extreme depression cohort. Among subdomains, enjoyment of life and social activities exhibited the largest increases (1.03 and 0.98 hSDS, respectively), while household and familial activities, essential daily activities, leisure and recreation, sleep, and work increased by 0.94, 0.87, 0.86, 0.81, and 0.79 hSDS. The overall pattern indicates a global rather than domain-specific deterioration of daily functioning. These depression-related worsening were paralleled by a corresponding deterioration of the functional capacity and assessed with the FFbHR, which deteriorated by up to 0.94 hSDS for patients with extreme depression.

CPGS subscores showed hSDS scores for patients with extreme depression of 0.87 for pain-related interference with social/familial/leisure activities, 0.83 for interference with daily life activities, 0.81 for interference with work/housework, and 0.77 for the number of days impaired by pain in the last three months.

Mental and affective indices showed the steepest gradients. The VR-12 MCS deteriorated by 2.66 hSDS across depression categories, representing the strongest single effect observed in the dataset. Similarly, the Marburg Questionnaire on Habitual Health Findings (MQHHF) increased by 1.38 hSDS, indicating worsening general well-being. The affective dimension of the pain sensation scale increased to 1.57 hSDS, and the sensory dimension to 1.15 SDS, suggesting progressive amplification of both sensory and emotional components of pain perception. FABQ scores, both for work and physical activity, worsened similarly and reached 0.84 and 0.57 hSDS, respectively. In contrast to that, the VR-12 PCS worsened only moderately (0.49 hSDS), showing that the mental and affective domains were more strongly affected than purely physical aspects.

As did all parameters before, the PDQ7 score also increased continuously with depression severity, from 0.00 to 0.93 hSDS, indicating a shift toward neuropathic or mixed pain patterns among more depressed patients.

The Pain Self-Efficacy Questionnaire (PSEQ) revealed a striking, inverse pattern relative to all other measures. Mean hSDS values increased from 0.00 to 1.19 hSDS across depression categories, representing the most pronounced deterioration among all non-psychological scales. On the original scale, this translated into an average reduction of more than 13.1 points (33.5%) between non-depressed and extremely depressed patients, with a corresponding increase in the proportion of individuals below the critical threshold of 25.6 points from 11.5% to 51.7%.

**Figure 7:**
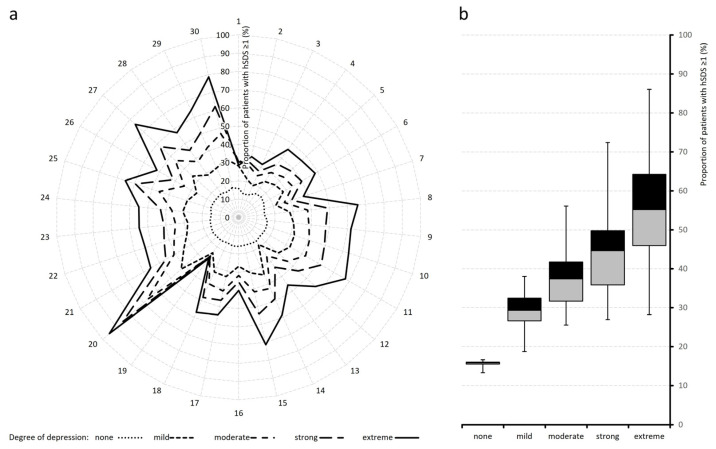
Distribution of harmonized standardized deviation scores (hSDS ≥ 1) across depression severity categories. Radar (spider) plot (**left**, **a**) showing the proportion (%) of patients with harmonised standard deviation scores (hSDS) > 1 across 30 items, stratified by degree of depression (none, mild, moderate, strong, extreme). Each axis represents one item, and values increase radially from the center. Line styles indicate depression severity. Higher severity levels are consistently associated with a greater proportion of patients exceeding the >1 SDS threshold, with pronounced peaks in specific items (e.g., around items 20 and 26–30). Corresponding boxplots (**right**, **b**) illustrating the distribution of the proportion of patients with hSDS > 1 across depression severity groups (none to extreme). Boxes represent the interquartile range (IQR), medians are indicated by horizontal lines, and whiskers show variability. Numerical item descriptions correspond to the list of evaluated parameters in Table 1 and Table 2.

**Figure 8:**
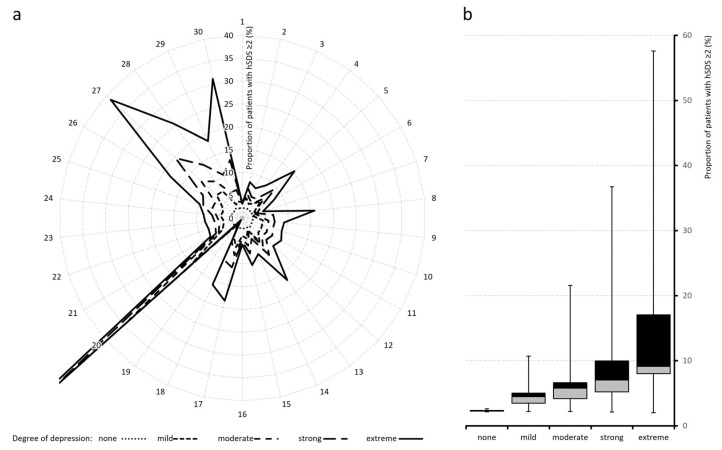
Distribution of harmonized standardized deviation scores (hSDS ≥ 2) across depression severity categories. Radar (spider) plot (**left**, **a**) showing the proportion (%) of patients with harmonised standard deviation scores (hSDS) > 2 across 30 items, stratified by degree of depression. Compared to the >1 SDS threshold, proportions are generally lower but display a similar pattern, with higher depression severity associated with increased proportions and distinct peaks in selected items (notably around item 20 and the upper item range). Corresponding boxplots (**right**, **b**) depicting the distribution of the proportion of patients with hSDS > 2 across depression severity groups. The overall proportions are lower than for the >1 SDS threshold, but a clear gradient remains, with increasing depression severity associated with higher proportions and greater variability. Numerical item descriptions correspond to the list of evaluated parameters in Table 1 and Table 2.

Table 3 and Figure 7 and Figure 8 detail the proportions of patients whose hSDS values exceeded thresholds of ≥1 and ≥2, representing severe and extreme deteriorations relative to the reference group.

At hSDS ≥ 1, only 15.6% of patients in the non-depressed cohort were affected, compared with an average of 56.6% in the extreme depression group. For hSDS ≥ 2, proportions increased from 2.3% to ~14.7%. The steepest hSDS ≥ 1 rises occurred in mental quality of life (VR-12 MCS; 95.1%), affective pain processing (76.1%), pain-related disability (61.7 to 71.4%), overall well-being (65.1%), and self-efficacy, which together accounted for nearly two-thirds of high-deterioration cases.

### 3.4. Pain-Related Self-Efficacy

Aggregated information on self-efficacy and its impairment by depression is given in Table 4. The proportion of patients with a clinically relevant impairment (defined as PSEQ ≤ 25.6) was 51.7% of patients with extreme depression, 36.6% with strong depression, 26.3% with moderate depression, 18.4% with mild depression, and 11.5% without depression. Corresponding odds ratios/relative risks vs. no depression increased from 1.7/1.1 for mild depression to 2.7/1.2 for moderate, 4.4/1.4 for strong, and 8.2/1.8 for extreme depression.

The average (median) hSDS increased from 0.0 (0.0) for none to 0.41 (0.42) for mild, 0.65 (0.78) for moderate, 0.94 (0.96) for strong, and 1.19 (1.31) for extreme depression and was accompanied by a significant increase (*p* < 0.001 for all!) in the proportion of patients with hSDS ≥ 1/2: 15.3/2.2% for no depression, 29.6/3.2% for mild, 42.0/6.1% for moderate, 50.9/10.3% for strong, and 64.0/18.5% for extreme depression. Corresponding odds ratios vs. no depression increased from 2.3/1.4 for mild to 4.0/2.8 for moderate, 5.8/5.1 for strong, and 9.8/9.9 for extreme depression.

### 3.5. Domain-Level Correlations and Multivariate Structure Analysis

Pairwise Pearson correlations among SDS-transformed variables demonstrated highly consistent patterns across biopsychosocial domains. Pain intensity measures showed strong intercorrelations (r = 0.78–0.94), and pain-related disability indices were closely linked to functional limitations (r = 0.72–0.89). Measures of psychological distress, including affective pain processing, mental quality of life (VR-12 MCS), and habitual well-being (MQHHF), exhibited the strongest associations with depression severity (r values generally >0.70). Self-efficacy (PSEQ) was inversely associated with nearly all symptom measures, with the strongest negative correlations observed with mental quality of life (r = −0.68), pain disability (r = −0.63), and depressive symptom severity (r = −0.60), supporting its role as a cross-domain construct.

An exploratory PCA of standardized SDS profiles indicated that a single latent severity dimension accounted for 84% of total variance, suggesting that the majority of measured constructs change coherently with increasing depression. A secondary component capturing an affective–self-efficacy contrast explained an additional 9% of variance. Variables with the strongest loadings on this secondary component included PSEQ self-efficacy (negative loading), affective pain sensitivity, VR-12 MCS, and MQHHF well-being, indicating that emotional adaptation processes differentiate patients beyond global symptom escalation.

These multivariate patterns were consistent with the directional relationships evaluated in the path models and further supported a conceptual separation between functional–physical burden, psychological–affective distress, and self-efficacy as a bridging construct.

### 3.6. PCA-Informed Path Analysis: Bottom-Up and Top-Down Frameworks

**Figure 9:**
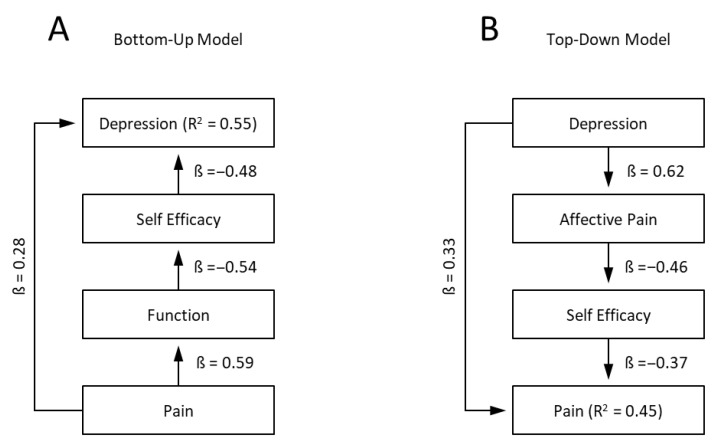
Mediation models illustrating pain ↔ depression pathways. Left panel (**A**): Bottom-up structural model illustrating the relationships between pain intensity (Pain), functional limitation (Function), self-efficacy, and depressive symptoms (Depression). Values represent standardized path coefficients (β). The model explains 55% of the variance in depression (R^2^ = 0.55). Right panel (**B**): Top-down structural model illustrating the relationships between depressive symptoms (Depression), affective pain processing (Affective Pain), self-efficacy (Self Efficacy), and pain intensity (Pain). Values represent standardized path coefficients (β). The model explains 45% of the variance in pain intensity (R^2^ = 0.45).

Based on the multivariate correlation and PCA findings indicating a dominant severity component and a secondary affective–self-efficacy axis, two complementary structural path models were estimated to evaluate directional relationships among pain, function, depression, and self-efficacy (see Figure 9A,B). In the bottom-up model (pain → function → self-efficacy → depression), the overall model explained 55% of the variance in depression scores (R^2^ = 0.55; *p* < 0.001). Direct effects of pain on depression were moderate (β = 0.28; *p* < 0.001), whereas indirect pathways mediated by functional limitation and self-efficacy accounted for the majority of explained variance (β_indirect = 0.41). Self-efficacy contributed the largest unique share (β = −0.48; *p* < 0.001), underscoring its cross-domain role as a psychological mediator between somatic and affective burden.

In the top-down model (depression → affective pain → self-efficacy → pain intensity), 45% of the variance in pain intensity was explained (R^2^ = 0.45; *p* < 0.001). Depression exerted strong direct effects on affective pain processing (β = 0.62; *p* < 0.001) and an indirect effect on pain via reduced self-efficacy (β_indirect = 0.33; *p* < 0.001). Across both frameworks, inclusion of age, sex, and BMI as covariates did not materially alter the significance or magnitude of associations, confirming the robustness of the model structure independent of demographic influences.

Arrows indicate standardized regression weights (β), with R^2^ values reflecting explained variance in outcome domains. Age, sex, and BMI were included as covariates; their effects were non-significant and are omitted from the path diagram for clarity.

### 3.7. Sensitivity and Robustness Checks

To evaluate robustness, all mediation analyses were repeated after excluding patients with extreme depression (n = 24,093). The indirect effects and path coefficients remained stable (variations < 5%), confirming that results were not driven by outliers or ceiling effects. Additional models adjusting for age, sex, and BMI showed minimal attenuation of path strength, indicating limited demographic confounding.

Collinearity diagnostics (VIF < 3.0 for all predictors) confirmed acceptable independence among predictors prior to PCA and path modeling, and residual analysis showed normally distributed errors without heteroscedasticity.

## 4. Discussion

This large-scale analysis of over 147,000 chronic pain patients from the German Pain e-Registry provides compelling, quantitative evidence for the deeply intertwined nature of pain, function, depression, and self-efficacy. Across all domains, hSDS worsened systematically with the degree of depressive symptom severity, indicating a robust, dose-dependent relationship between mood and biopsychosocial function. Importantly, while pain and disability rose in parallel with depression, self-efficacy declined sharply and consistently, emerging as the single most influential psychological factor mediating the bidirectional relationship between pain and mood. Although the path models are framed directionally, they are not intended to imply temporal causality. Given the cross-sectional design, they should be interpreted as theory-informed representations of dependency structures rather than as proof of causal or mechanistic pathways. Accordingly, the bottom-up and top-down frameworks reflect conceptually plausible mechanisms that are statistically compatible with the observed data, but they do not establish temporal order or direction of effect. In this context, hSDS values are intended to facilitate comparative interpretation across domains and depression strata and should not be understood as absolute clinical scores or diagnostic thresholds.

These findings extend previous observations that depression and chronic pain are mutually reinforcing conditions [1,2,3,4,5,10,14]. However, the present study moves beyond simple comorbidity descriptions by quantifying the proportional contribution of self-efficacy and showing that this variable bridges the physical and affective dimensions of chronic pain. Self-efficacy not only mediated the influence of pain and disability on depressive symptoms (bottom-up model) but also explained a large part of the reverse, top-down effect of depression on pain intensity through affective pain processing. This pattern delineates a reciprocal feedback loop in which low self-efficacy amplifies both emotional distress and pain perception, while restoring self-efficacy may simultaneously attenuate both.

### 4.1. The Graded Relationship Between Depression and Pain-Related Functioning

The stepwise deterioration across depression categories supports a continuous rather than categorical model of the pain–depression relationship. Even mild depressive symptoms were associated with measurable worsening in pain intensity (0.2 hSDS) and function (0.3–0.4 hSDS), suggesting that mood alterations exert clinically meaningful effects long before diagnostic thresholds for major depression are met. This is in line with the biopsychosocial model of pain, which conceptualizes emotional and cognitive processes as intrinsic components of the pain experience rather than external comorbidities [6,12].

The monotonic increase in both sensory and affective pain dimensions (SES-S and SES-A) further indicates that depression alters not only the subjective appraisal of pain but possibly its central modulation. Functional imaging studies have demonstrated overlapping neural circuits for pain and affect regulation—particularly within the anterior cingulate cortex, insula, and prefrontal areas—which are dysregulated in chronic pain and major depressive disorder [15,16,17]. These convergent mechanisms explain why pain becomes more intense, diffuse, and emotionally distressing as depression worsens, even in the absence of peripheral nociceptive change.

Moreover, the rise in PDQ7 scores with increasing depression severity suggests that neuroplastic changes associated with chronic mood disturbance may contribute to a shift toward neuropathic-like pain patterns. Although the PDQ7 is phenomenological rather than diagnostic, the near-linear increase from nociceptive to mixed or neuropathic presentations underscores the neuropsychological transformation of pain under depressive conditions—a finding with direct implications for mechanism-based treatment selection.

### 4.2. Functional Impairment and the Erosion of Quality of Life

The modified Pain Disability Index (mPDI) and its seven subdomains showed broad deterioration, confirming that chronic pain and depression jointly restrict nearly every aspect of daily life. The strongest effects were observed for enjoyment of life and social participation, highlighting the social isolation that often accompanies both pain and mood disorders. The VR-12 Mental Component Summary (MCS) displayed the steepest single gradient (2.66 hSDS), indicating that emotional well-being deteriorates far more rapidly than physical functioning (VR-12 PCS 0.49 hSDS). This imbalance mirrors the clinical observation that psychological suffering often outweighs physical impairment in determining overall quality of life in chronic pain populations.

The profound worsening in general well-being (MQHHF 1.38 hSDS) confirms this trend. Together, these data emphasize that addressing functional limitation and psychological distress must be regarded as primary treatment goals rather than secondary outcomes in pain management. The magnitude of deterioration observed here exceeds that typically reported in controlled trials, underlining the importance of large-scale real-world data to capture the full extent of chronic pain burden.

### 4.3. Multivariate Structure Supports a Three-Domain Biopsychosocial Model

Exploratory multivariate analyses provided additional support for the tripartite organization of pain-related symptom burden. Pairwise correlations revealed strong internal coherence within pain intensity and functional limitation measures, while psychological well-being, affective pain processing, and mental quality of life formed a distinct affective domain. Self-efficacy demonstrated substantial inverse associations across both clusters, consistent with its theorized role as a trans-domain resilience factor rather than a mere psychological consequence of pain.

Although clustering and PCA were exploratory due to the use of aggregated SDS profiles, results converged on a dominant latent severity dimension, which explained most of the variance across symptom domains, and a secondary affective–self-efficacy axis distinguishing patients with comparable symptom severity but different coping and adaptation profiles. This pattern aligns with prior evidence suggesting that self-efficacy modulates both the affective interpretation of nociceptive input and behavioral adaptation to persistent symptoms and reinforces the rationale for targeting self-efficacy therapeutically. This structural positioning mirrors its conceptual role in the fear–avoidance model of pain, in which beliefs about control and capability determine whether pain leads to recovery or chronicity [22].

### 4.4. Implications for Antidepressant Therapy in Pain Management

One of the most clinically relevant implications of this study concerns the interpretation of antidepressant efficacy in chronic pain. Traditionally, antidepressants—especially tricyclics and serotonin–norepinephrine reuptake inhibitors (SNRIs)—have been prescribed as co-analgesics, based on their ability to modulate descending inhibitory pathways and neurotransmitter balance [47,48,49]. However, the current data suggest an additional, potentially important mechanism: enhancement of self-efficacy.

If depression reduces perceived control and self-management ability, and if self-efficacy mediates the majority of the pain–depression interaction, then antidepressant treatment may be associated with improvements in pain outcomes through potential changes in self-efficacy-related processes. Several recent publications reported that effective antidepressant therapy improves pain coping, activity engagement, and treatment adherence even when pain intensity changes minimally [50,51]. These behavioral shifts correspond closely to the pattern observed in our path models, where self-efficacy accounted for 48% of the indirect variance between pain and depression.

Thus, the therapeutic rationale for antidepressants in chronic pain should extend beyond “co-analgesia” to encompass psychological empowerment—helping patients regain agency over their condition. This interpretation is consistent with neurobiological evidence suggesting that antidepressants are associated with normalization of prefrontal-limbic connectivity and cognitive control processes that underpin self-regulation [52].

Emerging neuroimaging data suggest that self-efficacy is not a purely psychological construct but is instantiated in a distributed fronto-limbic network. Core regions include the medial prefrontal cortex (mPFC), which integrates performance feedback and self-referential evaluation; the precuneus, which has been associated with trait-like general self-efficacy through structural plasticity; and the amygdala, which encodes motivational salience and emotional learning. These regions substantially overlap with neural circuits implicated in both chronic pain and major depression, particularly within prefrontal–limbic and default mode network pathways.

Antidepressants are known to modulate these systems, restoring top-down regulatory control from the mPFC over limbic reactivity and normalizing affective appraisal of somatic signals. From this perspective, the present mediation findings can be interpreted as reflecting a shared neurobiological framework: By influencing fronto-limbic circuitry, antidepressants may be associated with changes in perceived agency and coping capacity, which in turn may reduce affective pain amplification and functional disengagement. Integrating pharmacological and behavioral strategies that specifically target self-efficacy may therefore be a game changer for multidisciplinary pain treatment [53].

### 4.5. Bottom-Up and Top-Down Mechanisms: Two Sides of the Same Loop

The dual-framework path analyses provide robust quantitative evidence supporting a bidirectional interplay between somatic, functional, and affective domains in chronic pain. Both models confirmed that self-efficacy functions as the pivotal mediator linking physical and psychological aspects of the pain experience.

In the bottom-up model (*Pain → Function → Self-Efficacy → Depression*), reduced self-efficacy emerged as the key mediator translating physical dysfunction into emotional distress. Although pain exerted a direct effect on depression, the indirect pathway via functional limitation and reduced self-efficacy explained the majority of variance (R^2^ = 0.55). This underscores that loss of perceived control and confidence in coping—rather than pain intensity alone—drives depressive comorbidity in chronic pain. Clinically, this implies that strategies restoring a sense of mastery and competence may yield larger emotional benefits than focusing solely on analgesia.

Conversely, the top-down model (*Depression → Affective Pain → Self-Efficacy → Pain Intensity*) highlights the reciprocal mechanism by which depressive mood enhances pain perception and reduces coping ability. Here, depression strongly interferes with affective pain processing and self-efficacy, which together predicted the perceived intensity of pain (R^2^ = 0.45). This model supports a bi-directional, circular dynamic: depression exacerbates pain through cognitive-emotional channels, while pain perpetuates depressive symptoms through loss of control and helplessness. Such mutual amplification provides a mechanistic basis for the chronicity and treatment resistance often observed in comorbid pain and depression.

Together, the two models converge on the conclusion that self-efficacy acts as a psychological bridge between bottom-up (pain-driven) and top-down (mood-driven) mechanisms. This insight carries direct therapeutic implications: Interventions that restore or strengthen self-efficacy—such as cognitive-behavioral therapy, graded activity exposure, or motivational enhancement—may simultaneously attenuate depressive symptoms and reduce pain-related disability, even when analgesic effects are modest. These findings also suggest a broader role for antidepressants in pain management, not only as pharmacologic co-analgesics but as modulators of self-efficacy and affective resilience. By improving mood regulation and perceived coping capacity, such treatments could help interrupt the self-perpetuating feedback loop between pain and depression. Self-efficacy–oriented interventions may include structured self-management training, graded activity exposure, CBT-based coping enhancement, and pharmacological strategies that stabilize affect regulation.

Across both frameworks, inclusion of age, sex, and BMI as covariates did not materially alter the model structure, suggesting that these effects are robust and independent of demographic characteristics. Taken together, the models converge on the concept that self-efficacy acts as a psychological bridge between somatic dysfunction and affective distress, governing both bottom-up and top-down pathways in the chronic pain cycle.

The bidirectional strength of these pathways also explains why purely biomedical treatments often fail to achieve durable relief unless accompanied by psychological interventions such as cognitive-behavioral therapy (CBT), acceptance and commitment therapy (ACT), or self-management programs that explicitly reinforce efficacy beliefs [54,55,56].

### 4.6. Comparison with Previous Research

Previous studies have explored individual links between pain, depression, and self-efficacy, but few have analyzed them jointly in such a large and systematically harmonized dataset. Smaller observational studies (n < 500) have reported correlations between PSEQ scores and depression ranging from −0.45 to −0.60 [18,19,57], closely matching the present results. Randomized trials of self-management interventions in fibromyalgia and low-back pain have demonstrated improvements in self-efficacy that mediate both mood and functional gains [57,58].

What distinguishes the current analysis is its harmonized standardized deviation approach (hSDS), which normalizes effect sizes across instruments and domains. This allowed cross-domain comparability that is rarely achievable in clinical datasets, highlighting the uniform gradient of deterioration across pain, function, and mood. The large sample size also provided sufficient power to detect non-linear effects and to perform cluster- and PCA-based path analyses that validate theoretical models in real-world data.

### 4.7. Methodological and Conceptual Significance

Methodologically, this study illustrates the power of harmonized, patient-reported registry data such as those provided by the GPeR here for understanding complex biopsychosocial relationships. The exclusive use of PRMs avoids rater bias and ensures that observed associations reflect patients’ lived experience rather than external classification systems. By aligning score directions and standardizing deviations relative to a clinically relevant reference group (non-depressed patients), the hSDS approach transforms heterogeneous scales into a coherent analytic metric.

Conceptually, these findings reinforce the view that chronic pain should be regarded as a multisystem adaptation disorder involving affective, cognitive, and behavioral dimensions. Self-efficacy sits at the center of this adaptive system, mediating the transition between somatic input and emotional response. In this sense, self-efficacy can be conceptualized as the *psychological immune system* of the pain experience—buffering external stressors, maintaining functional stability, and modulating the subjective impact of pain.

### 4.8. Clinical and Research Implications

These findings have significant clinical implications. They suggest that therapeutic strategies aiming to enhance self-efficacy—including cognitive-behavioral therapy, acceptance-based interventions, or structured self-management programs—may interrupt the vicious circle linking pain and depression.

Importantly, the data may provide a new conceptual rationale for the use of antidepressants in chronic pain. Rather than viewing them exclusively as pharmacologic co-analgesics, their potential role in modulating mood, coping, and perceived self-efficacy could represent a paradigm shift in pain management. Even in the absence of direct analgesic effects, improving self-efficacy and emotional regulation may yield tangible benefits in pain coping and overall functioning.

Furthermore, this integrative modeling approach provides a framework for future longitudinal studies to assess causal dynamics and treatment effects. By quantifying mediation through self-efficacy, it becomes possible to measure psychological treatment targets objectively and to evaluate whether improvements in this construct precede reductions in pain and depressive symptoms.

## 5. Strengths and Limitations

The present study benefits from a very large sample size, standardized documentation, and harmonization procedures that ensured comparability across diverse assessment instruments. By normalizing all variables according to the non-depressed reference cohort, the analyses captured relative deviations in standardized effect sizes, thus enabling meaningful cross-domain comparisons.

Nevertheless, some limitations should be considered.

First, the cross-sectional design precludes causal inference, and the models, although conceptually directional, cannot confirm temporal order. Second, although all instruments were validated patient-reported measures, clinical or physician-rated data—such as diagnostic subtypes or treatment histories—were not included. Therefore, residual confounding by variables such as medication use, pain duration, or psychiatric comorbidity cannot be excluded and represents an inherent limitation of registry-based PRM-only analyses. Third, DASS-21 (the tool used to separate study cohorts) captures current symptom severity, rather than providing a formal clinical diagnosis according to ICD-10/11 or DSM-5 criteria. Consequently, misclassification between transient distress and clinically relevant depressive disorders cannot be fully excluded. However, the continuous nature of the DASS-21 scores aligns with the conceptual framework of the present study, which sought to model depression as a dimensionally graded correlate of pain and function, rather than a categorical comorbidity. Fourth, while the PDQ-7 allowed for a phenomenological classification of nociceptive versus neuropathic pain profiles, specific diagnostic categories were not available. As a result, this analysis could not differentiate between distinct pain conditions such as neuropathic, nociceptive, or mixed pain syndromes. However, this design feature also represents a conceptual strength within the context of this study. The aim was to model the biopsychosocial relationships among pain, function, self-efficacy, and depression across rather than within diagnostic categories. By analyzing pain as a multifactorial and transdiagnostic phenomenon, the results more closely reflect the heterogeneity encountered in real-world chronic pain populations. Nevertheless, diagnosis-specific differences in the magnitude or structure of these associations cannot be excluded. Future studies should therefore integrate both patient-reported and diagnostic data to examine whether the identified bottom-up and top-down pathways vary by pain type or underlying mechanism. Fifth, multivariate structure analyses were exploratory due to the use of aggregated hSDS means, and findings should therefore be interpreted as supportive rather than confirmatory. Sixth, although large datasets increase statistical power, they may also amplify minor associations that have limited clinical relevance. Seventh, because the analysis was restricted to complete cases, a degree of selection bias cannot be excluded. Patients with missing patient-reported measures may differ systematically from those included, which may limit generalizability. And finally, as the data derive from a German registry embedded in a specific health-care system, cultural and system-level factors may limit generalizability to other settings.

Nonetheless, the convergence of findings across multiple scales and analytic strategies supports the robustness of the observed relationships.

## 6. Conclusions

This comprehensive analysis of more than 147,000 chronic pain patients highlights a highly coherent pattern of associations between pain intensity, functional impairment, depressive symptom burden, and pain-related self-efficacy. Within the applied exploratory, cross-sectional models, self-efficacy consistently occupied a central statistical position linking somatic and affective domains across both bottom-up (pain-driven) and top-down (mood-driven) frameworks.

The findings suggest that self-efficacy functions as a key bridging construct within the biopsychosocial organization of chronic pain, modulating the strength of associations between physical dysfunction and emotional distress. Exploratory multivariate structure analyses further supported a conceptual separation between somatic burden, affective distress, and adaptive coping capacity, with self-efficacy positioned at their intersection.

Importantly, these results are hypothesis-generating and do not imply causal or mechanistic relationships. All directional and mediation models should be interpreted as statistical representations of cross-sectional associations rather than as evidence of temporal or causal effects. However, they provide a coherent conceptual framework for future longitudinal and interventional research. In this context, interventions that aim to enhance self-efficacy—pharmacological or non-pharmacological—may represent promising strategies to address both depressive symptoms and pain-related disability as part of integrated, multimodal pain management approaches.

## 7. Plain Language Summary

Chronic pain and depression often occur together and can strongly affect daily functioning and quality of life. In this study, we analyzed data from more than 147,000 people with chronic pain recorded in the German Pain e-Registry to better understand how pain, emotional distress, and coping-related factors are related to each other.

We found consistent patterns showing that people with more severe depressive symptoms also reported higher pain-related impairment and lower confidence in their ability to manage pain (self-efficacy). Statistical models suggested that self-efficacy occupies a central position in the relationships between pain, functional limitations, and depressive symptoms.

Importantly, this study does not show cause-and-effect relationships. The analyses describe associations observed at one point in time and are intended to generate hypotheses for future research. However, the findings suggest that supporting patients’ confidence in managing their condition may be an important focus for future pain treatment strategies and research.

## Figures and Tables

**Table 1:** Descriptive statistics of pain, function, and quality-of-life measures across DASS-21 depression categories.

			Degree of Depression					Degree of Depression					Degree of Depression				
		All	None	Mild	Moderate	Strong	Extreme	None	Mild	Moderate	Strong	Extreme	None	Mild	Moderate	Strong	Extreme
DASS-21 cut-offs (point ranges)		-	0–4	5–6	7–10	11–13	14–21	0–4	5–6	7–10	11–13	14–21	0–4	5–6	7–10	11–13	14–21
	Patients (n)	147,217	50,611	20,793	33,916	17,804	24,093	50,611	20,793	33,916	17,804	24,093	50,611	20,793	33,916	17,804	24,093
	(%)	100.0	34.4	14.1	23.0	12.1	16.4	34.4	14.1	23.0	12.1	16.4	34.4	14.1	23.0	12.1	16.4
Parameter (dimension, measure)/number			**Original (raw) data**					**Harmonized absolute difference to “none”**					**Harmonized relative difference to “none”**				
Age (years, mean)	1	55.9	56.2	56.6	56.0	55.4	55.1	0.0	0.4	0.2	0.8	1.1	0.0	0.9	0.5	1.8	2.5
Body mass index (kg/m^2^, mean)	2	27.7	27.2	27.4	27.8	28.0	28.6	0.0	0.2	0.6	0.8	1.4	0.0	0.7	2.2	2.9	5.1
Tailored treatment target (TTT; mm VAS, mean)	3	21.8	19.4	20.8	22.2	23.6	26.0	0.0	1.4	2.8	4.2	6.6	0.0	1.7	3.5	5.2	8.2
24-h. pain intensity index (PIX; mm VAS, mean)	4	52.9	47.5	51.3	53.5	56.7	61.7	0.0	3.8	6.0	9.2	14.2	0.0	7.2	11.4	17.5	27.0
Lowest 24-h. pain intensity (LPI; mm VAS, mean)	5	29.1	23.5	27.1	29.7	33.1	38.6	0.0	3.6	6.2	9.6	15.1	0.0	4.7	8.1	12.5	19.7
Average 24-h. pain intensity (API; mm VAS, mean)	6	52.9	46.7	51.2	54.0	57.4	62.7	0.0	4.5	7.3	10.7	16.0	0.0	8.4	13.7	20.1	30.0
Highest 24-h. pain intensity (HPI; mm VAS, mean)	7	75.6	70.1	74.1	76.6	79.6	83.9	0.0	4.0	6.5	9.5	13.8	0.0	13.4	21.7	31.8	46.2
Modified pain disability index (mPDI; mm VAS, mean)	8	54.5	44.5	51.9	56.6	61.8	69.1	0.0	7.4	12.1	17.3	24.6	0.0	13.3	21.8	31.2	44.3
Disability of household and familial activities (mPDI #1; mm VAS, mean)	9	56.9	46.9	54.5	59.1	64.0	71.5	0.0	7.6	12.2	17.1	24.6	0.0	14.3	23.0	32.2	46.3
Disability of leisure and relaxation (mPDI #2; mm VAS, mean)	10	59.9	50.3	57.8	61.9	66.9	73.6	0.0	7.5	11.6	16.6	23.3	0.0	15.1	23.3	33.4	46.9
Disability of social activities (mPDI #3; mm VAS, mean)	11	57.1	45.7	54.5	59.6	65.6	73.7	0.0	8.8	13.9	19.9	28.0	0.0	16.2	25.6	36.6	51.6
Disability of work (business or household; mPDI #4; mm VAS, mean)	12	62.5	53.1	60.5	64.8	69.5	75.8	0.0	7.4	11.7	16.4	22.7	0.0	15.8	24.9	35.0	48.4
Disability of essential daily activities (mPDI #5; mm VAS, mean)	13	35.3	26.2	32.0	36.6	42.0	50.4	0.0	5.8	10.4	15.8	24.2	0.0	7.9	14.1	21.4	32.8
Disability of sleep (mPDI #6; mm VAS, mean)	14	48.8	39.2	46.0	50.8	56.0	63.0	0.0	6.8	11.6	16.8	23.8	0.0	11.2	19.1	27.6	39.1
Disability of enjoyment of life (mPDI #7; mm VAS, mean)	15	60.0	48.9	57.7	62.6	68.2	75.4	0.0	8.8	13.7	19.3	26.5	0.0	17.2	26.8	37.8	51.9
Fear-avoidance beliefs—work (FABQ; NRS 42, mean)	16	16.9	13.0	16.1	18.2	19.5	22.1	0.0	3.1	5.2	6.5	9.1	0.0	10.7	17.9	22.4	31.4
Fear-avoidance beliefs—physical activity (FABQ; NRS 24, mean)	17	13.4	11.7	13.4	14.0	14.6	15.5	0.0	1.7	2.3	2.9	3.8	0.0	13.8	18.7	23.6	30.9
Functional capacity (FFbHR; NRS 100, mean)	18	60.1	68.3	61.8	58.8	54.0	47.5	0.0	6.5	9.5	14.3	20.8	0.0	9.5	13.9	20.9	30.5
Physical quality-of-life (VR12 PCS; NRS 100, mean)	19	31.2	33.5	31.1	30.4	29.7	28.5	0.0	2.4	3.1	3.8	5.0	0.0	7.2	9.3	11.3	14.9
Mental quality-of-life (VR12 MCS; NRS 100, mean)	20	42.3	52.2	44.8	39.6	34.4	28.8	0.0	7.4	12.6	17.8	23.4	0.0	14.2	24.1	34.1	44.8
Days in past 3 months where usual activities were prevented because of pain (CPGS #4; NRS 92, mean)	21	47.3	37.1	45.1	49.4	54.6	62.4	0.0	8.0	12.3	17.5	25.3	0.0	12.7	19.6	27.8	40.2
Pain interference with daily activities in the last 3 months (CPGS #5; mm VAS, mean)	22	54.6	45.5	52.2	56.5	61.2	68.5	0.0	6.7	11.0	15.7	23.0	0.0	12.3	20.2	28.8	42.2
Pain interference with social/family/leisure activities in the last 3 months (CPGS #6; mm VAS, mean)	23	62.6	52.4	60.5	65.0	70.1	77.2	0.0	8.1	12.6	17.7	24.8	0.0	17.0	26.5	37.2	52.1
Pain Interference with work/housework in the last 3 months (CPGS #7; mm VAS, mean)	24	64.6	54.5	62.4	67.2	72.1	78.8	0.0	7.9	12.7	17.6	24.3	0.0	17.4	27.9	38.7	53.4
Overall well-being (MQHHF; NRS 35, mean)	25	14.9	18.7	15.1	13.7	12.6	10.4	0.0	3.6	5.0	6.1	8.3	0.0	19.3	26.7	32.6	44.4
Pain phenotype (PDQ7; NRS 35, mean)	26	13.8	11.4	13.0	14.2	15.5	17.6	0.0	1.6	2.8	4.1	6.2	0.0	6.8	11.9	17.4	26.3
Affective pain perception (SES-A; NRS 42, mean)	27	19.7	13.8	17.9	20.8	23.8	29.0	0.0	4.1	7.0	10.0	15.2	0.0	14.5	24.8	35.5	53.9
Sensory pain perception (SES-S; NRS 42, mean)	28	13.5	10.5	12.4	13.8	15.6	19.0	0.0	1.9	3.3	5.1	8.5	0.0	6.0	10.5	16.2	27.0
Pain-related self-efficacy (PSEQ; NRS, mean)	29	33.9	39.7	35.1	32.4	29.2	26.4	0.0	4.6	7.3	10.5	13.3	0.0	11.6	18.4	26.4	33.5
Quality-of-life impairment by pain (QLIP; NRS 40, mean)	30	15.9	20.4	17.0	14.9	12.6	9.5	0.0	3.4	5.5	7.8	10.9	0.0	16.7	27.0	38.2	53.4
Mean	-	41.9	37.6	40.7	42.7	44.9	48.3	0.0	5.0	8.0	11.4	16.3	0.0	11.3	17.9	25.3	36.0
Standard Deviation (SD)	-	18.9	17.1	18.4	19.6	21.3	23.9	0	2.6	4.2	6.0	8.3	0	5.0	7.8	10.7	14.6
Sum	30	1255.7	1128.1	1221.3	1280.7	1347.3	1450.3	0.0	149.0	239.0	341.4	487.8	0.0	337.8	537.1	758.3	1079.0
95% CI	-	0.1	0.2	0.3	0.2	0.4	0.3	na	0.0	0.1	0.1	0.1	na	0.1	0.1	0.2	0.2

Values represent raw means (left section) as well as harmonized absolute (middle), and relative differences (right) versus the reference cohort without depression (“none”). Data are based on validated patient-reported outcomes from the German Pain e-Registry. Higher harmonized scores indicate greater impairment/deterioration.

**Table 2:** Harmonized standardized deviation scores (hSDSs) across pain, function, psychological, and self-efficacy measures.

		Degree of Depression					Degree of Depression					Degree of Depression				
		None	Mild	Moderate	Strong	Extreme	None	Mild	Moderate	Strong	Extreme	None	Mild	Moderate	Strong	Extreme
DASS-21 cut-offs (point ranges)		0–4	5–6	7–10	11–13	14–21	0–4	5–6	7–10	11–13	14–21	0–4	5–6	7–10	11–13	14–21
	Patients (n)	50,611	20,793	33,916	17,804	24,093	50,611	20,793	33,916	17,804	24,093	50,611	20,793	33,916	17,804	24,093
	(%)	34.4	14.1	23.0	12.1	16.4	34.4	14.1	23.0	12.1	16.4	34.4	14.1	23.0	12.1	16.4
Parameter number		**hSDS (reference “none”; mean)**					**hSDS (reference “none”; SD)**					**hSDS (reference “none”; 95% CI)**				
Age (years, mean)	1	0.00	0.03	0.01	0.05	0.07	1.01	0.99	0.97	0.94	0.92	0.01	0.02	0.01	0.02	0.01
Body mass index (kg/m^2^, mean)	2	0.00	0.03	0.10	0.14	0.24	1.01	1.07	1.10	1.14	1.21	0.01	0.02	0.01	0.02	0.02
Tailored treatment target (TTT; mm VAS, mean)	3	0.00	0.09	0.18	0.27	0.43	1.00	0.99	1.03	1.05	1.15	0.01	0.02	0.01	0.02	0.02
24-h. pain intensity index (PIX; mm VAS, mean)	4	0.00	0.21	0.34	0.52	0.80	1.00	0.95	0.92	0.91	0.89	0.01	0.01	0.01	0.02	0.01
Lowest 24-h. pain intensity (LPI; mm VAS, mean)	5	0.00	0.19	0.33	0.51	0.79	1.00	1.02	1.04	1.08	1.15	0.01	0.02	0.01	0.02	0.02
Average 24-h. pain intensity (API; mm VAS, mean)	6	0.00	0.22	0.36	0.52	0.78	1.00	0.95	0.92	0.92	0.89	0.01	0.01	0.01	0.02	0.01
Highest 24-h. pain intensity (HPI; mm VAS, mean)	7	0.00	0.18	0.30	0.44	0.63	1.00	0.90	0.84	0.79	0.70	0.01	0.01	0.01	0.01	0.01
Modified pain disability index (mPDI; mm VAS, mean)	8	0.00	0.34	0.55	0.79	1.12	1.00	0.92	0.89	0.89	0.90	0.01	0.01	0.01	0.01	0.01
Disability of household and familial activities (mPDI #1; mm VAS, mean)	9	0.00	0.29	0.47	0.65	0.94	1.00	0.93	0.90	0.90	0.89	0.01	0.01	0.01	0.02	0.01
Disability of leisure and relaxation (mPDI #2; mm VAS, mean)	10	0.00	0.28	0.43	0.61	0.86	1.00	0.92	0.90	0.89	0.90	0.01	0.01	0.01	0.02	0.01
Disability of social activities (mPDI #3; mm VAS, mean)	11	0.00	0.31	0.49	0.70	0.98	1.00	0.94	0.92	0.91	0.90	0.01	0.01	0.01	0.02	0.01
Disability of work (business or household; mPDI #4; mm VAS, mean)	12	0.00	0.26	0.41	0.57	0.79	1.00	0.93	0.89	0.87	0.85	0.01	0.01	0.01	0.01	0.01
Disability of essential daily activities (mPDI #5; mm VAS, mean)	13	0.00	0.21	0.37	0.57	0.87	1.00	1.01	1.03	1.04	1.06	0.01	0.02	0.01	0.02	0.02
Disability of sleep (mPDI #6; mm VAS, mean)	14	0.00	0.23	0.39	0.57	0.81	1.00	0.99	0.98	0.97	0.97	0.01	0.02	0.01	0.02	0.01
Disability of enjoyment of life (mPDI #7; mm VAS, mean)	15	0.00	0.34	0.53	0.75	1.03	1.00	0.91	0.89	0.90	0.94	0.01	0.01	0.01	0.02	0.01
Fear-avoidance beliefs—work (FABQ; NRS 42, mean)	16	0.00	0.29	0.48	0.60	0.84	1.00	1.02	1.02	1.06	1.11	0.01	0.02	0.01	0.02	0.02
Fear-avoidance beliefs—physical activity (FABQ; NRS 24, mean)	17	0.00	0.25	0.34	0.43	0.57	0.99	0.91	0.88	0.87	0.88	0.01	0.01	0.01	0.01	0.01
Functional capacity (FFbHR; NRS 100, mean)	18	0.00	0.29	0.43	0.65	0.94	1.00	1.01	1.01	1.06	1.05	0.01	0.02	0.01	0.02	0.02
Physical quality-of-life (VR12 PCS; NRS 100, mean)	19	0.00	0.24	0.30	0.37	0.49	1.00	0.88	0.82	0.75	0.68	0.01	0.01	0.01	0.01	0.01
Mental quality-of-life (VR12 MCS; NRS 100, mean)	20	0.00	0.84	1.43	2.02	2.66	0.99	1.04	1.04	0.94	0.89	0.01	0.02	0.01	0.02	0.01
Days in past 3 months where usual activities were prevented because of pain (CPGS #4; NRS 92, mean)	21	0.00	0.24	0.37	0.53	0.77	1.00	1.00	0.98	0.97	0.92	0.01	0.02	0.01	0.02	0.01
Pain interference with daily activities in the last 3 months (CPGS #5; mm VAS, mean)	22	0.00	0.24	0.40	0.57	0.83	1.00	0.95	0.93	0.91	0.88	0.01	0.01	0.01	0.02	0.01
Pain interference with social/family/leisure activities in the last 3 months (CPGS #6; mm VAS, mean)	23	0.00	0.29	0.44	0.62	0.87	1.00	0.92	0.87	0.83	0.78	0.01	0.01	0.01	0.01	0.01
Pain Interference with work/housework in the last 3 months (CPGS #7; mm VAS, mean)	24	0.00	0.26	0.42	0.58	0.81	1.00	0.91	0.86	0.81	0.74	0.01	0.01	0.01	0.01	0.01
Overall well-being (MQHHF; NRS 35, mean)	25	0.00	0.60	0.83	1.02	1.38	1.00	0.78	0.74	0.72	0.80	0.01	0.01	0.01	0.01	0.01
Pain phenotype (PDQ7; NRS 35, mean)	26	0.00	0.24	0.42	0.61	0.93	1.00	1.00	1.01	1.04	1.07	0.01	0.02	0.01	0.02	0.02
Affective pain perception (SES-A; NRS 42, mean)	27	0.00	0.42	0.72	1.03	1.57	1.00	1.04	1.05	1.05	1.00	0.01	0.02	0.01	0.02	0.01
Sensory pain perception (SES-S; NRS 42, mean)	28	0.00	0.26	0.45	0.69	1.15	1.00	1.08	1.14	1.20	1.33	0.01	0.02	0.01	0.02	0.02
Pain-related self-efficacy (PSEQ; NRS, mean)	29	0.00	0.41	0.65	0.94	1.19	1.00	0.92	0.91	0.85	0.87	0.01	0.01	0.01	0.01	0.01
Quality-of-life impairment by pain (QLIP; NRS 40, mean)	30	0.00	0.50	0.81	1.15	1.60	0.99	0.93	0.90	0.86	0.83	0.01	0.01	0.01	0.01	0.01
Mean	-	0.00	0.28	0.45	0.64	0.92										
Standard Deviation (SD)	-	1.00	0.15	0.25	0.34	0.45										
Median	-	0.00	0.26	0.42	0.59	0.85										
Minimum	-	0.00	0.03	0.01	0.05	0.07										
Maximum	-	0.00	0.84	1.43	2.02	2.66										
95%-CI	-	na	0.06	0.10	0.14	0.18										
Significance vs. “none”	-	na	<0.001	<0.001	<0.001	<0.001										
Effect size vs. “none”	-	na	0.288	0.465	0.662	0.944										

Mean hSDS values (left section), standard deviations (middle), and 95% confidence intervals (right) are shown for each DASS-21 depression category. hSDS normalization was performed relative to the non-depressed reference group.

**Table 3:** Proportion of patients with clinically relevant hSDSs (≥1 and ≥2) by depression category.

		Degree of Depression					Degree of Depression				
		None	Mild	Moderate	Strong	Extreme	None	Mild	Moderate	Strong	Extreme
DASS-21 cut-offs (point ranges)		0–4	5–6	7–10	11–13	14–21	0–4	5–6	7–10	11–13	14–21
	Patients (n)	50,611	20,793	33,916	17,804	24,093	50,611	20,793	33,916	17,804	24,093
	(%)	34.4	14.1	23.0	12.1	16.4	34.4	14.1	23.0	12.1	16.4
Parameter number		**Proportion of patients with hSDS ≥ 1 (%)**					**Proportion of patients with hSDS ≥ 2 (%)**				
Age (years, mean)	1	15.80	28.07	31.34	30.32	28.59	2.20	3.47	3.46	3.12	3.24
Body mass index (kg/m^2^, mean)	2	13.02	21.58	28.58	30.52	33.98	2.22	4.40	5.43	6.47	8.07
Tailored treatment target (TTT; mm VAS, mean)	3	13.55	19.01	24.92	27.58	31.70	2.27	3.39	4.23	5.02	7.14
24-h. pain intensity index (PIX; mm VAS, mean)	4	16.05	24.38	30.35	36.03	46.00	2.36	3.97	4.01	5.24	8.88
Lowest 24-h. pain intensity (LPI; mm VAS, mean)	5	16.60	26.66	33.00	38.17	46.42	2.28	5.40	6.53	9.47	15.34
Average 24-h. pain intensity (API; mm VAS, mean)	6	15.85	28.12	34.09	39.80	48.29	2.48	3.14	3.39	4.56	7.95
Highest 24-h. pain intensity (HPI; mm VAS, mean)	7	14.67	21.48	26.30	30.54	37.29	2.10	2.54	2.97	3.39	4.77
Modified pain disability index (mPDI; mm VAS, mean)	8	14.14	28.13	38.01	48.86	65.61	2.46	2.72	3.94	6.77	15.86
Disability of household and familial activities (mPDI #1; mm VAS, mean)	9	15.75	30.24	38.67	47.91	61.72	2.35	4.51	5.77	7.15	9.21
Disability of leisure and relaxation (mPDI #2; mm VAS, mean)	10	16.17	32.06	40.68	49.40	63.31	2.31	4.58	5.81	7.06	9.04
Disability of social activities (mPDI #3; mm VAS, mean)	11	15.92	32.35	42.21	52.22	67.40	2.31	4.69	6.13	7.58	9.78
Disability of work (business or household; mPDI #4; mm VAS, mean)	12	15.60	29.11	35.94	43.84	56.54	2.50	4.66	5.76	7.02	9.06
Disability of essential daily activities (mPDI #5; mm VAS, mean)	13	16.29	18.36	26.05	33.86	45.80	2.64	6.36	7.63	9.97	16.71
Disability of sleep (mPDI #6; mm VAS, mean)	14	15.75	34.48	42.56	48.81	58.39	2.21	2.81	3.37	4.94	8.61
Disability of enjoyment of life (mPDI #7; mm VAS, mean)	15	15.59	31.01	41.63	54.01	71.35	2.29	4.56	6.11	7.93	10.48
Fear-avoidance beliefs—work (FABQ; NRS 42, mean)	16	15.87	26.91	31.80	35.34	40.09	2.29	3.88	4.59	5.10	5.78
Fear-avoidance beliefs—physical activity (FABQ; NRS 24, mean)	17	15.93	33.08	41.18	46.46	54.53	2.21	6.51	8.27	11.01	18.47
Functional capacity (FFbHR; NRS 100, mean)	18	15.19	32.69	39.80	47.98	56.92	2.17	4.54	5.63	9.93	15.91
Physical quality-of-life (VR12 PCS; NRS 100, mean)	19	15.88	24.00	26.68	26.18	27.79	2.29	1.77	1.30	0.88	0.50
Mental quality-of-life (VR12 MCS; NRS 100, mean)	20	15.87	42.28	67.31	85.06	95.14	2.31	14.57	33.23	57.68	80.71
Days in past 3 months where usual activities were prevented because of pain (CPGS #4; NRS 92, mean)	21	16.33	34.60	40.90	45.99	55.49	2.36	5.00	5.91	6.65	8.02
Pain interference with daily activities in the last 3 months (CPGS #5; mm VAS, mean)	22	15.86	29.73	37.09	42.79	53.66	2.28	4.27	5.33	6.15	7.71
Pain interference with social/family/leisure activities in the last 3 months (CPGS #6; mm VAS, mean)	23	15.65	27.94	34.72	41.14	54.64	2.34	4.18	5.19	6.15	8.17
Pain Interference with work/housework in the last 3 months (CPGS #7; mm VAS, mean)	24	15.02	30.68	36.70	42.95	54.88	2.34	4.78	5.72	6.69	8.55
Overall well-being (MQHHF; NRS 35, mean)	25	16.06	29.51	45.70	59.35	65.14	2.42	4.45	6.89	8.94	9.82
Pain phenotype (PDQ7; NRS 35, mean)	26	16.02	26.51	34.32	41.25	51.64	2.46	5.07	6.84	9.89	18.10
Affective pain perception (SES-A; NRS 42, mean)	27	15.84	33.77	46.57	57.95	76.06	2.27	7.51	12.02	19.61	38.71
Sensory pain perception (SES-S; NRS 42, mean)	28	16.17	28.64	37.70	45.42	57.32	2.58	7.28	9.94	14.36	25.55
Pain-related self-efficacy (PSEQ; NRS, mean)	29	15.30	29.56	41.95	50.94	63.96	2.23	3.23	6.10	10.34	18.47
Quality-of-life impairment by pain (QLIP; NRS 40, mean)	30	16.62	32.71	46.90	62.10	78.61	2.25	3.70	6.33	12.98	31.09
Mean	-	15.70	29.21	37.99	45.78	56.63	2.33	4.73	6.59	9.40	14.66
Standard Deviation (SD)	-	0.67	4.88	8.27	11.60	14.03	0.12	2.24	5.35	9.66	14.64
Sum	31	468.38	867.65	1123.65	1342.75	1648.25	69.78	141.95	197.84	282.08	439.69
95%-CI	-	na	2.00	3.38	4.75	5.74	na	0.92	2.19	3.95	5.99

Frequencies and percentages refer to the share of patients exceeding each hSDS threshold for individual scales and composite domains.

**Table 4:** Pain-related self-efficacy (PSEQ) and derived indicators across depression categories.

Degree of Depression (DASS-21 Categories)	None	Mild	Moderate	Strong	Extreme
(DASS-21 range)	0–4	5–6	7–10	11–13	14–21
Number of patients (n)	50,611	20,793	33,916	17,804	24,093
(%)	(34.4)	(14.1)	(23.0)	(12.1)	(16.4)
Pain-related self-efficacy [PSEQ; raw data [mean (SD)]	39.7 (11.2)	35.1 (10.3)	32.4 (10.2)	29.2 (9.5)	26.4 (9.8)
[median]	40	35	31	29	25
[range]	10–60	10–60	10–60	10–60	10–57
[95% CI]	0.112	0.160	0.124	0.160	0.141
Patients with PSEQ < 25.63 (n)	5839	3824	8920	6522	12,444
(%)	(11.5)	(18.4)	(26.3)	(36.6)	(51.7)
Odds ratio vs. none	na	1.728	2.736	4.433	8.191
(95% CI)	na	(1.653–1.806)	(2.638–2.838)	(4.255–4.618)	(7.892–8.501)
Relative risk vs. none	na	1.084	1.200	1.396	1.830
(95% CI)	na	(1.076–1.092)	(1.192–1.209)	(1.38–1.412)	(1.805–1.854)
Effect size vs. none	na	0.091	0.191	0.286	0.436
Significance vs. none	na	<0.001	<0.001	<0.001	<0.001
Pain-related self-efficacy [PSEQ; hSDS [mean (SD)]	0.00 (1.00)	0.41 (0.92)	0.65 (0.91)	0.94 (0.85)	1.19 (0.87)
[median]	−0.03	0.42	0.78	0.96	1.31
[range]	−1.81–2.65	−1.81–2.65	−1.81–2.65	−1.81–2.65	−1.54–2.65
[95% CI]	0.010	0.014	0.011	0.014	0.013
Effect size vs. none	na	0.426	0.677	1.009	1.26
Significance vs. none	na	<0.001	<0.001	<0.001	<0.001
Patients with hSDS scores ≥ 1 (n)	7745	6146	14,228	9068	15,410
(%)	(15.3)	(29.6)	(42)	(50.9)	(64)
Odds ratio vs. none	na	2.322	4.000	5.745	9.823
(95% CI)	na	(2.235–2.413)	(3.872–4.132)	(5.53–5.968)	(9.478–10.18)
Relative risk vs. none	na	1.202	1.459	1.726	2.350
(95% CI)	na	(1.191–1.214)	(1.445–1.473)	(1.7–1.753)	(2.31–2.391)
Effect size vs. none	na	0.164	0.298	0.363	0.492
Significance vs. none	na	<0.001	<0.001	<0.001	<0.001
Patients with hSDS scores ≥ 2 (n)	1131	672	2069	1841	4449
(%)	(2.2)	(3.2)	(6.1)	(10.3)	(18.5)
Odds ratio vs. none	na	1.461	2.842	5.046	9.908
(95% CI)	na	(1.326–1.61)	(2.64–3.06)	(4.675–5.445)	(9.263–10.598)
Risk ratio vs. none	na	1.010	1.041	1.090	1.199
(95% CI)	na	(1.007–1.013)	(1.038–1.044)	(1.085–1.096)	(1.192–1.206)
Effect size vs. none	na	0.029	0.099	0.174	0.289
Significance vs. none	na	<0.001	<0.001	<0.001	<0.001

## Data Availability

The data are not publicly available due to registry governance and data protection regulations. Aggregated or summary data may be made available from the corresponding author upon reasonable request and with permission of the GPeR steering committee.
